# Spatiotemporal Modeling of Premature Mortality in Mississippi: A County-Level Analysis of Years of Potential Life Lost, 2015–2025

**DOI:** 10.5888/pcd23.260035

**Published:** 2026-08-13

**Authors:** Jae Eun Lee, Junghye Sung, Ji-Young Lee, Yalanda Barner, Edith Offiah, Alyce Hays

**Affiliations:** 1 College of Health Sciences, Jackson State University, Jackson, Mississippi; 2 University of South Florida, Sarasota, Florida

## Abstract

**Introduction:**

Understanding premature mortality through years of potential life lost (YPLL) is essential for identifying preventable deaths and health inequities, yet county-level spatiotemporal patterns remain understudied in states such as Mississippi that have high rates of premature mortality. This study examined spatiotemporal trends in premature mortality across Mississippi’s 82 counties (2015–2025) using years of potential life lost (YPLL) before age 75 years.

**Methods:**

We determined county-level YPLL rates from County Health Rankings & Roadmaps (2015–2025) (underlying deaths from approximately 2012 to 2023). Trends were compared with US averages, stratified by education, income, and insurance status; mapped via GIS; analyzed for spatial clustering with calculated local indicators of spatial association (LISA); and modeled by using spatial autoregressive (SAR) lag models with year fixed effects.

**Results:**

From 2015 to 2025, Mississippi’s YPLL rate rose 35.2% (10,918.3 to 14,763.9 per 100,000), far outpacing national trends and widening the gap between the state and the US by approximately 50%. Contrary to expectation, the largest increases occurred in high-education, high-income, low-uninsured (mostly urban/coastal) counties. Hot spots intensified in rural Delta and Mississippi River counties, while cold spots disappeared from urban and coastal areas. SAR models showed strong spatial dependence (ρ ≈ 0.32); key drivers were injury-related deaths (β = 58.9, *P* < .001), percentage of population that is non-Hispanic Black, and diabetes prevalence, while higher median household income was associated with lower YPLL.

**Conclusion:**

Mississippi’s premature mortality pattern has shifted: rural burdens persist, but rapid deterioration in urban and more affluent counties now drives statewide worsening. Effective policy requires sustained rural investment in chronic disease control along with urban-focused interventions targeting injury and violence prevention, trauma systems, and mental health/substance-use services.

**Summary**

**What is already known on this topic?**

Mississippi has premature mortality rates 20% to 30% above national averages, driven primarily by chronic disease and socioeconomic disadvantages concentrated in rural Delta counties.

**What is added by this report?**

This first spatiotemporal county-level analysis (2015–2025) showed that premature mortality rates in Mississippi have worsened and shifted: rural hot spots intensified while former urban/coastal cold spots disappeared, producing the largest disparities in socioeconomically advantaged counties and strong spatial dependence dominated by injury-related deaths.

**What are the implications for public health practice?**

Mississippi requires a dual-track strategy: continued rural investment in chronic disease control along with urban/coastal interventions in violence prevention, trauma systems, and behavioral health services, with data-driven geographic targeting to reverse the state’s accelerating burden of premature mortality.

## Introduction

Years of potential life lost (YPLL) before age 75 years is a public health metric that measures premature mortality by summing years lost when deaths occur before a reference age (1). Unlike crude or age-adjusted mortality rates, YPLL assigns greater weight to deaths occurring at younger ages (typically before age 75 years), thereby emphasizing preventable causes such as injuries, violence, and substance use that disproportionately affect working-age populations (2), with substantial burdens observed for causes such as opioid overdose (3). In contrast, age-adjusted mortality rates are particularly effective for comparing the burden of chronic diseases (eg, cardiovascular disease, cancer, diabetes) across populations with differing age structures; many such deaths occur after age 75 years. Because of its interpretability and focus on preventable early deaths, YPLL is particularly valuable for prioritizing interventions and quantifying health disparities (4,5).

Mississippi consistently ranks among the worst states for premature mortality, demonstrating a “southern mortality penalty” that translates to mortality rates 20% to 30% above national averages (6). As of 2024–2025, the state’s YPLL rate exceeded 14,000 per 100,000 population, far surpassing the US average of approximately 10,276 per 100,000 population; this rate was driven by cardiovascular disease, cancer, injuries, and widening racial inequities, particularly among non-Hispanic Black residents (7,8). Despite this persistent burden, no published study has applied a spatiotemporal analytic framework to examine county-level YPLL trends across Mississippi’s 82 counties. National and regional analyses have identified broad patterns (9–11), but they cannot inform state-specific resource allocation or reveal local shifts in clustering and drivers.

The primary objective of this study was to quantify spatiotemporal disparities and trends in YPLL across Mississippi counties from 2015 to 2025 by using data from County Health Rankings & Roadmaps (hereinafter, County Health Rankings). Secondary objectives included comparing state and national trajectories, identifying socioeconomic and demographic predictors of elevated YPLL, mapping evolving spatial clusters, and deriving policy recommendations for rural and urban contexts. By filling the gap in state-focused spatiotemporal analysis, this work provides actionable evidence for Mississippi policymakers responsible for health planning, budget allocation, and disparity reduction.

## Methods

### Data sources and measures

This secondary analysis used county-level data from the 2015 through 2025 releases of County Health Rankings (12,13), a program of the University of Wisconsin Population Health Institute that compiles indicators from multiple national sources, including National Center for Health Statistics mortality files (via CDC WONDER), the Behavioral Risk Factor Surveillance System, the US Census Bureau’s American Community Survey, and others. Detailed methodology and data sources are available elsewhere: www.countyhealthrankings.org/health-data/methodology-and-sources. Because the data for this study are publicly available and deidentified, institutional review board approval was not required.

The primary outcome was YPLL before age 75 per 100,000 population, sourced directly from County Health Rankings. YPLL estimates are calculated from underlying death records as 3-year rolling averages, with suppression applied when fewer than 10 deaths occur. Due to processing and reporting lags, the 2015–2025 releases reflect mortality occurring from approximately 2012 through 2023. We compared values from Mississippi’s 82 counties with contemporaneous national YPLL values from the same County Health Rankings releases.

Covariates included continuous county-level measures of health behaviors (eg, adult smoking, physical inactivity), clinical care (eg, uninsured rate, primary care physician ratio), social and economic factors (eg, median household income, high school graduation rate, children in poverty), and physical environment (eg, air pollution particulate matter, access to healthy foods). All variables were standardized across releases through harmonization of naming, units, and definitions to enable longitudinal analysis.

For subgroup analyses, we dichotomized 11 key socioeconomic indicators annually within each release year separately for Mississippi counties and all non-Mississippi US counties. We set cutpoints at the median of nonmissing values within each stratum, creating comparable “high” and “low” groups that accounted for temporal and distributional differences between Mississippi and the rest of the nation.

### GIS mapping and spatial analysis

We used Python’s geopandas and matplotlib libraries to conduct geographic information systems (GIS) mapping and visualize longitudinal spatial patterns of YPLL across Mississippi’s counties. To highlight geographic disparities, we generated choropleth maps with Jenks natural breaks classification (14). To assess spatial dependence and test for geographical clustering of YPLL rates, we used the global Moran *I*, implemented via the PySAL library’s esda module (15,16). We calculated local indicators of spatial association (LISA) (15) to identify high-YPLL and low-YPLL clusters, with significance set at *P* < .05. We defined spatial weights by using Queen contiguity to account for shared county boundaries, computed with PySAL’s libpysal module (17).

### Local spatial autocorrelation analysis

We calculated LISA for each year (2015–2025) by using Anselin Local Moran *I* to identify significant clusters of YPLL (15). We conducted year-specific analyses because the underlying 3-year rolling mortality averages provided stable estimates for annual inference.

We created Queen contiguity weights (shared border or vertex) and row-standardized these weights with libpysal. We assessed significance via 999 random permutations (*P* < .05). We classified counties as

High–high: counties with above-average YPLL surrounded by counties that also have above-average YPLL (hot spots)
Low–low: counties with below-average YPLL surrounded by counties with below-average YPLL (cold spots)
Low–high: counties with below-average YPLL surrounded by counties with above-average YPLL (spatial outliers)
High–low: counties with above-average YPLL surrounded by counties with below-average YPLL (spatial outliers)
Not significant: no significant clustering

We generated maps for each year in Python using geopandas, matplotlib, and contextily (CartoDB Positron basemap) with 2023 TIGER/Line county boundaries (500 k resolution). A pooled LISA analysis across all data from 2015 through 2025 produced nearly identical clustering patterns, confirming the robustness and temporal stability of the observed spatial disparities. We used pandas, geopandas, libpysal, and esda to perform all analyses.

### Spatial autoregressive (SAR) lag model with temporal fixed effects

To assess spatial and temporal clustering in premature mortality (measured as YPLL), we estimated a SAR lag model with year fixed effects (17,18):

yit=ρ∑jwijyjt+Xiβ+γt+ϵit,ϵit∼N(0,σ2)

where

- *y_it_* is the YPLL in county *i* at time *t* (2015–2025),
- ρ is the SAR coefficient,
- *w_ij_* are elements of a row-standardized Queen contiguity weights matrix *W* (shared boundary or vertex) constructed using libpysal,
- $\sum_{j}w_{ij}y_{jt}$ is the spatially lagged dependent variable (average YPLL in neighboring counties at time *t*),
- *X_i_* is a vector of time-invariant, standardized county-level covariates (adult smoking, diabetes prevalence, injury-related deaths, percentage non-Hispanic Black, percentage aged 65 or older, uninsured adults, median household income),
- β are regression coefficients,
- γ*_t_* are year-specific dummy variables (fixed effects) to capture temporal trends and shocks, with 2015 as the reference year, and
- ϵ_it_ is the idiosyncratic error.

The model was fit by using maximum likelihood estimation via the spreg.ML_Lag function in Python’s PySpatial package, with robust SEs. The spatial lag term accounts for endogenous spatial dependence, while year dummies flexibly model nonlinear temporal effects, including the abrupt mortality increase observed from 2021 through 2024. We tested the significance of ρ by using a likelihood ratio test against a nonspatial pooled ordinary least squares model with year fixed effects. We evaluated model performance by using pseudo *R* ^2^, Akaike Information Criterion, and Moran *I* on residuals to assess remaining spatial autocorrelation.

To provide a comprehensive spatiotemporal analysis of YPLL trends, we used complementary approaches: multilevel mixed-effects regression to compare longitudinal trajectories between Mississippi and non-Mississippi counties while accounting for county-level clustering; LISA to map and track the evolution of significant spatial clusters over time; and a SAR lag model with year fixed effects to quantify spatial dependence and identify key socioeconomic and health-related drivers of YPLL. Together, these methods build incrementally: multilevel models establish differential trends, LISA reveals changing geographic clustering patterns, and SAR lag models explain the underlying spatial processes and dominant predictors (eg, injury-related deaths).

### Multilevel and subgroup analyses

We modeled longitudinal YPLL trends by using mixed-effects linear regression (statsmodels, Python) with county-level random intercepts and fixed effects for year (linear and quadratic terms). We fitted separate models for Mississippi and non-Mississippi counties to compare trajectories.

Subgroup analyses examined disparities in the most recent period (2023–2025 releases). Using the 22 subgroups defined by the dichotomization procedure described previously, we calculated mean YPLL for Mississippi and non-Mississippi counties in each subgroup. We tested absolute differences with 2-sample Welch *t* tests (unequal variances) and reported effect sizes as Cohen *d.* All analyses were performed in Python 3.11 using pandas, numpy, and scipy.stats.

## Results

### Longitudinal trends in YPLL

County-level YPLL rates in Mississippi were consistently higher than the average national rates (Table 1). Mississippi’s YPLL rate increased from 10,918.3 per 100,000 in 2015 to 14,763.9 in 2025 (35.2% increase), compared with a national increase from 7,892.0 to 10,275.6 (30.2% increase). The gap widened from 3,026.3 in 2015 to 4,488.3 in 2025 (48.3% increase), with a temporary narrowing in 2019–2020 (2,932.6). Multilevel mixed-effects quadratic models confirmed Mississippi's steeper increase in YPLL compared with the rest of the US. In Mississippi (901 observations, 82 counties), the model estimated a baseline YPLL rate of 11,160.9 (SE, 263.1) per 100,000 (*P* < .001) with significant upward curvature (year_centered^2^: β = 56.0; 95% CI, 47.1–64.8; *P* < .001) and better quadratic fit (log-likelihood –7,799.1 vs –7,872.1 for linear). For non-Mississippi counties (32,980 observations, 3,027 counties), the quadratic coefficient was smaller (β = 28.9, *P* < .001; log-likelihood –285,100.0 vs –285,905.7 for linear), with likelihood ratio tests confirming superior quadratic fit in both (*P* < .001). Mississippi’s YPLL increased more rapidly than the national average, with a steeper upward trajectory during the study period. 

**Table 1:** Rates of YPLL in Mississippi and the US and Difference Between the Two Rates^a^

Year	2015	2016	2017	2018	2019	2020	2021	2022	2023	2024	2025
US	7,892.0	7,928.0	7959.9	8,072.0	8,404.2	8,455.2	8,416.9	8,814.1	8,814.1	9,737.0	10,275.6
Mississippi	10,918.3	11,013.0	11,063.6	11,377.2	11,447.7	11,387.8	11,521.5	12,519.7	12,519.7	14,020.7	14,763.9
Gap	3,026.3	3,085.1	3,103.7	3,305.1	3,043.5	2,932.6	3,104.5	3,705.7	3,705.7	4,283.7	4,488.3

Abbreviation: YPLL, years of potential life lost.

a Data source: County Health Rankings & Roadmaps (12,13).

### Subgroup analyses

Subgroup analyses from the 2023–2025 County Health Rankings releases showed consistently and significantly higher premature mortality in Mississippi counties compared with non-Mississippi counties across nearly all socioeconomic and demographic subgroups (Table 2). All 22 subgroups with sufficient sample size had significantly higher YPLL in Mississippi (all *P* < .001), with Cohen *d* ranging from 0.85 to 2.22 (median Cohen *d* = 1.26), indicating large to very large effect sizes. We observed the largest disparities for population characteristics that are typically protective in the US: percentage of children in poverty (low), median household income (high), percentage with some college education (high), percentage that is non-Hispanic Black (high), percentage aged 65 years or older (high), and percentage of children with no health insurance (low).

**Table 2:** Subgroup Analysis of Differences in YPLL Between Mississippi and Non-Mississippi Counties, 2023–2025^a^

Subgroup^b^	Mississippi		Non-Mississippi		Difference^c^	Cohen *d* d
	Mean (SD)	No. of counties	Mean (SD)	No. of counties		
Children in poverty (low)	12,187.0 (2,346.5)	125	7,640.4 (2,037.5)	4,528	4,546.6	2.22
Median household income (high)	12,290.0 (2,503.2)	123	7,769.8 (2,171.8)	4,500	4,520.2	2.07
Some college (high)	12,932.4 (3,083.7)	123	8,011.3 (2,472.1)	4,447	4,921.1	1.98
% Non-Hispanic Black (high)	15,162.5 (3,340.7)	123	10,001.1 (3,051.8)	4,559	5,161.3	1.69
% Population aged ≥65 y (high)	14,194.7 (2,533.7)	123	9,641.6 (3,060.6)	4,446	4,553.0	1.49
Children without health insurance (low)	14,358.1 (3,656.5)	123	9,298.9 (3,418.5)	4,562	5,059.2	1.48
Adults without health insurance (low)	13,078.4 (3,234.9)	123	8,636.2 (3,045.0)	4,515	4,442.3	1.46
Unemployment rate (low)	12,447.9 (2,429.0)	123	8,638.2 (2,984.3)	4,459	3,809.7	1.28
% Non-Hispanic White (high)	12,554.6 (2,463.4)	123	8,979.6 (2,830.9)	4,477	3,575.0	1.27
Unemployment rate (high)	15,088.3 (3,391.4)	123	10,560.6 (3,575.9)	4,553	4,527.7	1.27
% Population aged <18 y (low)	13,118.0 (2,724.8)	123	9,358.1 (2,998.7)	4,498	3,759.9	1.26
High school graduation (low)	14,324.3 (3,202.0)	134	9,733.0 (3,696.8)	5,492	4,591.2	1.25
High school graduation (high)	13,102.7 (3,143.8)	112	9,416.5 (2,967.0)	3,520	3,686.1	1.24
% Non-Hispanic White (low)	14981.6 (3,446.5)	123	10,231.2 (3,839.3)	4,535	4,750.4	1.24
% Population aged <18 y (high)	14,418.1 (3,555.1)	123	9,859.8 (3,801.8)	4,514	4,558.3	1.20
Children in poverty (high)	15,401.4 (3,206.3)	121	11,597.7 (3,411.0)	4,484	3,803.7	1.12
Median household income (low)	15,246.1 (3,200.7)	123	11,444.1 (3,479.0)	4,512	3,802.1	1.10
Uninsured adults (high)	14,457.8 (3,080.9)	123	10,586.6 (3,523.3)	4,497	3,871.2	1.10
% Aged ≥65 y (low)	13,341.5 (3,758.8)	123	9,578.0 (3,761.5)	4,566	3,763.5	1.00
Some college (low)	14,603.8 (3,162.0)	123	11,166.2 (3,522.9)	4,565	3,437.6	0.98
Children without health insurance (high)	13,178.1 (2,616.0)	123	9,927.7 (3,420.1)	4,450	3,250.4	0.96
% Non-Hispanic Black (low)	12,373.7 (2,415.6)	123	9,208.4 (3,742.8)	4,453	3,165.4	0.85

Abbreviation: YPLL, years of potential life lost.

a Data source: County Health Rankings & Roadmaps (12,13).

b “Low” and “high” categories represent county groupings based on the median value of each variable for a given year, with “low” indicating values below the median and “high” indicating values above the median.

c *P* value for all mean differences <.001.

d Cohen *d* is a standardized measure of effect size representing the magnitude of differences between groups in SD units. Conventional benchmarks are 0.2 (small), 0.5 (medium), and 0.8 (large); values >1.0 indicate very large effects.

### Choropleth mapping and LISA

**Figure 1:**
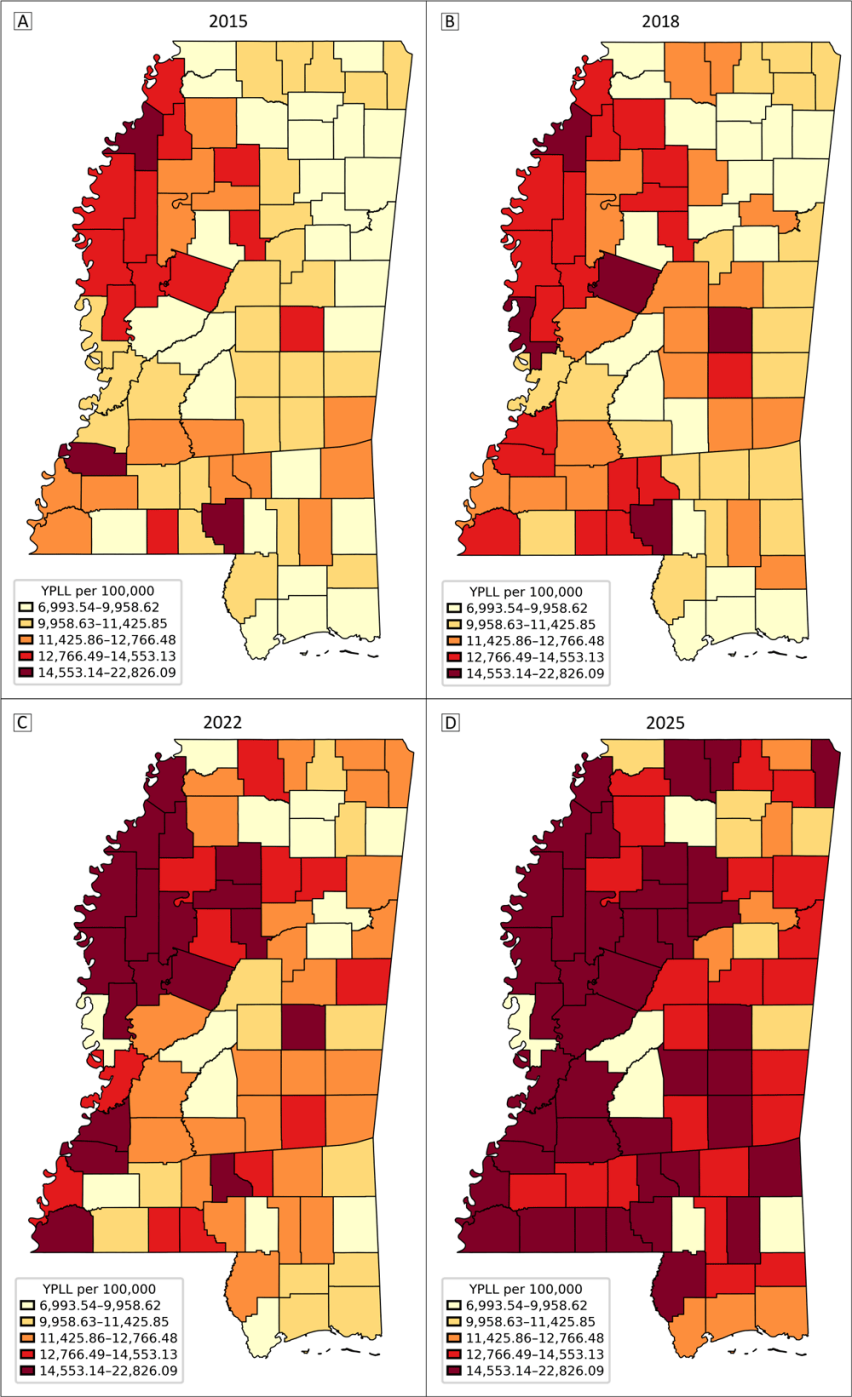
Choropleth map of years of potential life lost (YPLL) rates in A) 2015, B) 2018, C) 2022, and D) 2025, Mississippi. Choropleth maps were created by using quintile-based classification, with fixed cut points derived from the pooled distribution of county-level YPLL values across all study years to ensure consistent color assignment and comparability over time. Data source: 2015, 2018, 2022, 2025 releases of County Health Rankings and Roadmaps (12,13).

Choropleth maps showed evolving YPLL disparities, by quintile, across Mississippi’s 82 counties (Figure 1). In 2015, three counties were in the highest quintile of YPLL rates. By 2018, the Delta region (northwest) and counties along the Mississippi River in the southwest exhibited emerging high rates, with many falling into the highest and second-highest quintiles, while other areas showed an upward trend. In 2022, much of the Delta region reached the highest quintile. By 2025, most counties across the state were classified within the highest or second-highest quintiles of YPLL rates. Global Moran *I* showed significant spatial autocorrelation in 2015–2017 and 2021–2022 (*P* < .05) but weaker and nonsignificant autocorrelation in 2018–2020 and 2023–2025.

**Table:** 

Quintile YPLL	No. of counties, by year			
	2015	2018	2022	2025
6,993.54–9,958.62	27	20	14	6
9,958.63–11,425.85	27	20	13	6
11,425.86–12,766.48	14	19	26	7
12,766.49–14,553.13	12	19	12	23
14,553.14–22,826.09	3	5	18	41

**Figure 2:**
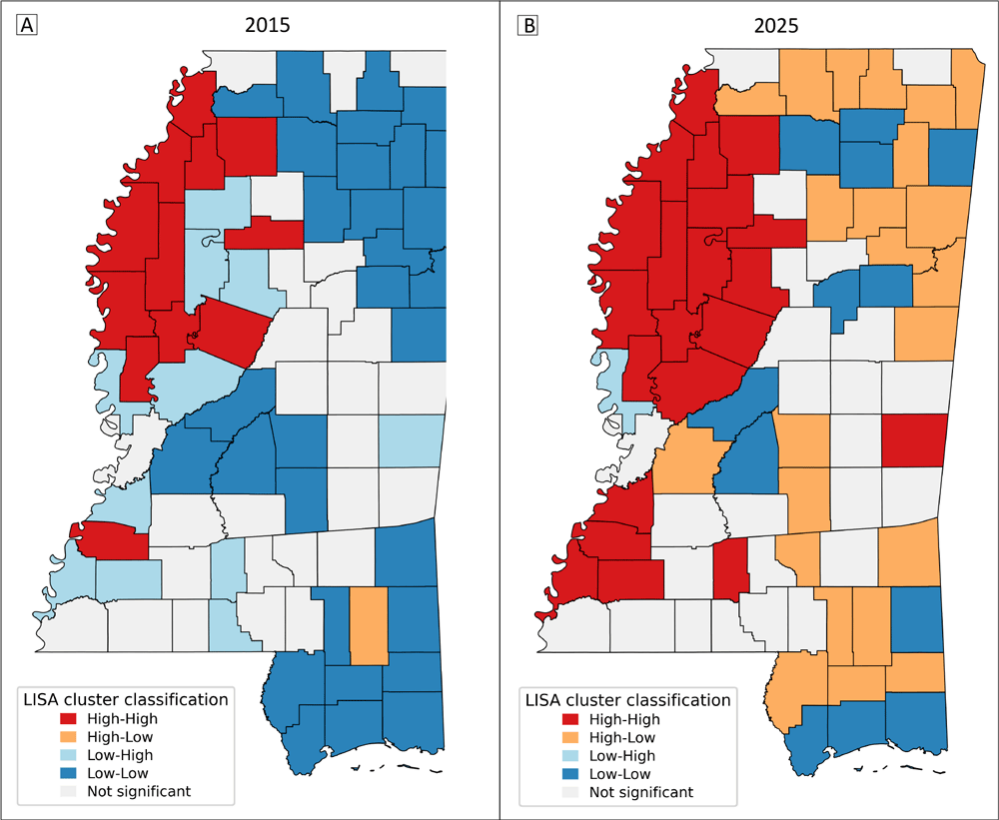
Local indicators of spatial association (LISA) cluster map of years of potential life lost (YPLL) rates in A) 2015 and B) 2025 in Mississippi. High–high indicates counties with above-average YPLL surrounded by counties with above-average YPLL; low–low indicates counties with below-average YPLL surrounded by counties with below-average YPLL; low–high indicates counties with below-average YPLL surrounded by counties with above-average YPLL; high–low indicates counties with above-average YPLL surrounded by counties with below-average YPLL; nonsignificant indicates areas with no significant clustering (*P* ≥ .05). Significance was based on 999 permutations (*P* < .05). Determinants of YPLL were from spatial autoregressive lag modeling. Data source: 2015 and 2025 release of County Health Rankings and Roadmaps (12,13).

LISA analysis identified significant clustering patterns across Mississippi (Figure 2). YPLL rates shifted markedly between the 2015 and 2025 County Health Rankings releases. High-high clusters increased from 12 counties in 2015 to 21 counties in 2025, with growth concentrated in the Delta and Mississippi River regions. By 2025, the following counties were added to the high–high category: Adams, Carroll, Claiborne, Franklin, Lauderdale, Lawrence, Leflore, Tallahatchie, and Yazoo counties. Meanwhile, the number of low–low clusters declined from 31 counties to 12, including several central and coastal counties, such as Hinds, Forrest, Monroe, and Chickasaw counties. This shift reflects a growing concentration of high YPLL rates in historically disadvantaged counties characterized by limited access to health care and higher levels of socioeconomic disadvantage, while previously low–low clusters (counties with below-average YPLL surrounded by counties with below-average YPLL), particularly in urban and coastal areas, transitioned to either nonclustered (*P* ≥ .05) or spatial outlier categories (low–high or high–low).

The SAR model showed a sharp, significant rise in YPLL starting in 2022 and continuing thereafter (2022: β = 1,676.80, *P* < .001; 2024: β = 3,017.40, *P* < .001; 2023: β = 1,713.12, *P* < .001; 2025: β = 3,457.57, *P* < .001) (Table 3).

**Table 3:** Regression Coefficients Determined by Spatial Autoregressive Lag Model With Additional Variables for Predictors of YPLL in Mississippi Counties, 2015–2025^a^^,^^b^

Variable	β Coefficient (SE)	*P* value
**Constant**	3,798.27 (1,333.83)	.004
**Year effects**		
2016	392.33 (223.03)	.08
2017	115.66 (229.18)	.61
2018	295.43 (257.76)	.25
2019	382.16 (274.31)	.16
2020	300.73 (272.60)	.27
2021	363.08 (285.98)	.20
2022	1,676.80 (271.61)	<.001
2023	1,713.12 (284.68)	<.001
2024	3017.4 (300.04)	<.001
2025	3,457.57 (325.74)	<.001
**Characteristics**		
Adult smoking	3,500.3 (2,408.96)	.15
Diabetes prevalence	9,124.93 (2,357.81)	<.001
Injury deaths	58.93 (2.77)	<.001
% Non-Hispanic Black	2,129.58 (395.94)	<.001
% Aged ≥65 y	−10,460.82 (2189.73)	<.001
Uninsured adults	−4,159.89 (2,587.97)	.11
Median household income	−0.07 (0.01)	<.001

Abbreviation: YPLL, years of potential life lost.

a Data source: County Health Rankings & Roadmaps (12,13).

b Dependent variable was YPLL; N = 901 observations (82 Mississippi counties × 11 years, 2015–2025); reference year was 2015 (omitted); spatial lag coefficient (ρ) = 0.32 (SE, 0.05); *P* < .001; pseudo *R* ^2^ = 0.749.

Significant predictors included injury-related deaths (β = 58.93, *P* < .001), diabetes prevalence (β = 9,124.93, *P* < .001), percentage of non-Hispanic Black residents (β = 2,129.58, *P* < .001), percentage of residents aged 65 years or older (β = −10,460.82, *P* < .001), and median household income (β = −0.07, *P* < .001). Adult smoking (β = 3,500.30, *P* = .15) and adults without health insurance (β = −4,159.89, *P* = .11) were not significant at conventional levels.

The spatial lag coefficient (ρ) from the SAR lag model was positive and significant (ρ = 0.32, *P* < .001), confirming strong spatial dependence: counties with elevated YPLL are clustered together, reinforcing geographic disparities. The model demonstrated strong explanatory power (pseudo *R* ^2^ = 0.749; N = 901 county-year observations).

## Discussion

This study documents a substantial and accelerating divergence in premature mortality between Mississippi and the rest of the US. From 2015 to 2025, according to County Health Rankings, Mississippi’s age-standardized YPLL rate increased 35.2%, from approximately 10,918 to 14,764 per 100,000, substantially outpacing the national rise of 30.2% and widening the state–US gap by nearly 50%. Multilevel mixed-effects models confirmed not only a steeper linear trend but also a pronounced quadratic acceleration in Mississippi, consistent with the disruptive effect of the COVID-19 pandemic and a simultaneous surge in injury-related deaths (19,20). These patterns are further shaped by structural inequities embedded in policy and social systems (21).

Subgroup analyses for the 2023–2025 releases showed that Mississippi counties had significantly higher YPLL across all 22 socioeconomic and demographic strata, with effect sizes ranging from large to very large (Cohen *d* > 0.8–1.5 for most). Strikingly, the largest absolute and relative excesses occurred in subgroups with characteristics traditionally considered protective: low percentage of children living in poverty, high median household income, high percentage with some college education, and low rates of uninsurance. These findings demonstrate a near-complete flattening of the social gradient in health in Mississippi — socioeconomic advantage confers dramatically less mortality protection than in the rest of the nation (22,23). At the same time, historically marginalized subgroups — non-Hispanic Black residents and adults aged 65 years or older — continued to bear extreme burdens, reflecting the enduring consequences of structural racism, residential segregation, and chronic disinvestment in the Mississippi Delta (24–26).

LISA analysis showed 2 concurrent and geographically contrasting phenomena. First, the number of high–high clusters, counties with above-average YPLL surrounded by counties that also have above-average YPLL, nearly doubled, from 12 to 21, consolidating along the rural Mississippi Delta and Mississippi River counties. These areas, among the poorest and most racially segregated in the US, have long had limited health care infrastructure, high levels of comorbidity (diabetes, hypertension, obesity), and, during 2020–2022, some of the nation’s highest COVID-19 case-fatality rates (21,26). Second, the number of low–low clusters (counties with below-average YPLL surrounded by counties with below-average YPLL) decreased from 31 to 12, as several urban and coastal counties, including Hinds (Jackson), Harrison (Gulfport–Biloxi), Rankin, and Forrest counties, transitioned from low–low clusters to either high–low (counties with above-average YPLL surrounded by below-average neighbors), low–high (counties with below-average YPLL surrounded by higher-YPLL neighbors), or nonclustered categories (*P* ≥ .05). This erosion of former bright spots signals an emerging urban mortality penalty driven predominantly by firearm-related homicide and suicide (disproportionately affecting young Black men), motor-vehicle fatalities, opioid and polysubstance overdoses, and lingering excess COVID-19 mortality among working-age adults.

The SAR lag model confirmed strong geographic dependence (ρ = 0.32, *P* < .001; pseudo *R* ^2^ = 0.749). Injury-related deaths are by far the strongest predictor (β = 58.93, *P* < .001), followed by diabetes prevalence (β = 9,124.93, *P* < .001) and percentage of non-Hispanic Black residents (β = 2,129.58, *P* < .001). Higher median household income (β = −0.07, *P* < .001) and a larger share of residents aged 65 or older (β = −10,460.82, *P* < .001) are associated with lower YPLL. Adult smoking and uninsured rates were not significant in the SAR lag model with year fixed effects. Year fixed effects for 2022–2025 showed a large pandemic-related increase in premature mortality, while the significant SAR lag term underscored spillover effects that amplified disparities across neighboring counties.

These findings converge on a key spatiotemporal shift: LISA analysis documented the intensification of rural high–high clusters and the near disappearance of urban/coastal low–low clusters, while the SAR lag model revealed strong geographic dependence (ρ = 0.32, *P* < .001) and identified injury-related deaths as the dominant driver. Together, these methods illustrate how long-standing disparities in rural areas have persisted and expanded, even as preventable injury-related urban penalties have eroded former bright spots (ie, areas with low rates of premature mortality) and accelerated the statewide rise in premature mortality.

### Strengths and limitations

Key strengths of this study include the integration of complementary longitudinal, spatial, and stratified analytic approaches in a single, fully reproducible open-source Python/Geopandas/PySAL workflow that can be readily updated with each annual release of the County Health Rankings. The use of 11 consecutive years of consistently defined policy-relevant county-level indicators permitted robust detection of both gradual trends and abrupt COVID-19–related shifts. The combination of multilevel trend models, detailed subgroup stratification across 22 socioeconomic and demographic strata, LISA analysis for cluster evolution, and a SAR model provided convergent evidence from multiple methodologic perspectives. Finally, the county-level resolution and explicit mapping of changing hot spots (high–high clusters) and cold spots (low–low clusters) offer actionable geographic targeting for interventions in Mississippi.

Despite these strengths, the study had several limitations primarily inherent to the County Health Rankings data ecosystem. First, YPLL and most covariates reflect deaths and conditions that occurred 2 to 4 years before the release year (eg, 2025 mortality estimates primarily capture data from 2021–2023), introducing a systematic lag that may underestimate the most recent surges in injury-related deaths or delay detection of potential reversals. Second, several behavioral and socioeconomic covariates (eg, adult smoking, uninsured rate) are model-based small-area estimates or periodic survey measures with varying reference periods and precision, potentially attenuating associations during periods of rapid change. Third, county-level aggregation obscures substantial within-county heterogeneity, especially in larger urban counties such as Hinds and Harrison. Fourth, the SAR specification assumes a single, homogeneous spatial dependence parameter (ρ) across the state, does not allow coefficients to vary spatially, and cannot simultaneously incorporate multilevel random effects for counties and years. Ongoing work is addressing this constraint by comparing the current SAR results with Bayesian hierarchical spatiotemporal models (eg, BYM2 and space–time separable specifications implemented in INLA and PyMC) that permit spatially varying coefficients, explicit county-level and year-level random effects, and fuller propagation of uncertainty: these approaches are expected to refine geographic targeting and uncertainty quantification in future iterations of this surveillance system. Fifth, although County Health Rankings provides a composite county-level measure of injury-related mortality, it does not disaggregate injury-related deaths into specific causes such as firearm homicide, suicide, motor-vehicle fatalities, or drug overdoses. This limitation prevents direct county-level decomposition within our dataset. However, state vital statistics and CDC WONDER data (27) consistently show substantial increases in drug overdose mortality (28,29) and firearm-related deaths (30) in Mississippi during 2018–2023, suggesting that these components likely contributed to the rising injury-related YPLL observed in our SAR lag model. Finally, as with any ecological analysis, individual-level causal inference is not possible.

### Policy implications

Mississippi can no longer rely on a monolithic rural-focused strategy. The Delta and River counties continue to require long-term investment in primary care expansion (especially federally qualified health centers), chronic disease management, broadband-enabled telehealth, and social determinants of health (housing, nutrition, education). Simultaneously, urban and coastal counties demand immediate, evidence-based interventions targeting the preventable drivers now dominating their increases in YPLL: hospital- and community-based violence interruption programs, focused deterrence, enhanced trauma systems, expansion of crisis response and mental health services, and comprehensive traffic safety measures. That even Mississippi’s most educated, insured, and affluent counties exceed national YPLL benchmarks underscores systemic failures, underfunded emergency and behavioral health infrastructure, racial inequities in service access, and insufficient political prioritization that blunt the protective effects seen elsewhere. Targeted, data-driven resource allocation guided by these spatial and subgroup findings is essential to reverse the state’s worsening trajectory.

### Conclusion

From 2015 to 2025, Mississippi transformed from a state with long-standing rural premature-mortality hot spots to one where preventable early death has diffused across virtually all socioeconomic strata and geographic settings. The persistence and intensification of Delta high–high clusters and the abrupt disappearance of urban/coastal low–low clusters illustrate how structural inequities and new preventable-death drivers, injury, violence, and behavioral health crises, interact with place. Reversing this crisis will require bold, equity-centered, and geographically nuanced policies that confront both historic disinvestment in rural Black communities and the emerging urban penalty now claiming thousands of additional potential years of life annually.
